# Partial object doubling in the periphery induced by negative afterimages

**DOI:** 10.1177/20416695241286787

**Published:** 2024-11-05

**Authors:** Ian M. Thornton, Anna Riga

**Affiliations:** Department of Cognitive Science, Faculty of Media and Knowledge Sciences, University of Malta, Msida, Malta

**Keywords:** visual illusions, negative afterimages, furrow illusion, local motion, global position

## Abstract

We describe a new phenomenon—partial object doubling—in which the perceived contours of peripherally viewed moving targets become distorted and duplicated. The effect appears to arise due to interactions between physically drawn contours and the strong negative afterimages that are dynamically released during stable viewing of the displays. An online demo is provided where the effect can be experienced and relevant parameters manipulated.

The purpose of this brief report is to describe a novel illusion in which clearly visible moving objects appear partially doubled when viewed peripherally. The effect can be directly experienced in Movie 1. During the first two cycles of the movie, the outline target circles embedded within the rectangular apertures are clearly visible. That is, all shape contours, together with their motion direction, can be veridically perceived, either with or without central fixation. However, when central fixation is maintained and the oriented inducing lines are added to the display, two illusory percepts become immediately apparent.

**Movie 1.**
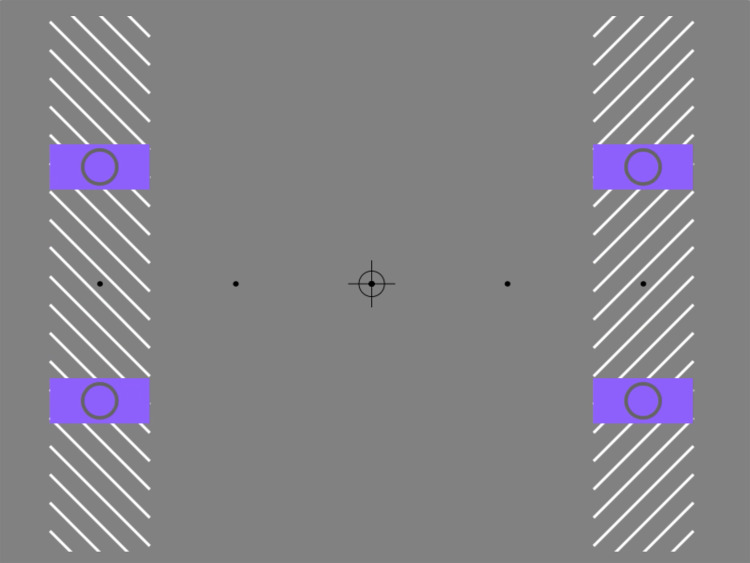
Partial object doubling. https://maltacogsci.org/POD/POD_Movie1.html

First, the moving elements no longer appear to track vertically up and down. Rather, they have an illusory diagonal trajectory, with the perceived angle jointly determined by the static orientation of the lines and the global direction of motion. Upwards-moving targets shift away from fixation, while downwards-moving targets shift towards fixation. This is a variant of the classic furrow illusion (Anstis, 2012; Cormack et al., 1992), and is thought to be due to the local motion signals generated by individual line occlusions being erroneously combined with the global motion of the entire aperture, an effect that only occurs in the periphery (Anstis, 2012).

The second—and novel—illusion relates to the central target shapes themselves. Instead of the percept of a single object within each moving rectangle—as seen during free viewing or in the absence of the inducing lines—now there is an apparent duplication of contours. The term that we think best describes this effect is “partial object doubling” (POD). The effect is extremely robust, with all observers who have seen the displays, either one-to-one or in large venues, being able to immediately experience the illusion, and to turn it on and off, simply by shifting their gaze between steady fixation and direct tracking of the targets.

The effect can be further explored online at https://maltacogsci.org/POD/, where a range of display parameters can be adjusted. The code for the demo and additional resources are also freely available on the Open Science Framework (OSF) page associated with this paper at https://osf.io/3q62d/.

What causes this illusory doubling? Our current explanation is that the doubling arises due to interactions between the drawn physical contours of the target shape and the strong negative afterimages that are dynamically released within the rectangular aperture in our version of the furrow illusion.

The presence of these afterimages can be more directly experienced in Movie 2, where we have alternated periods where the central target shape is present or absent. During the stable viewing required for all furrow-type illusions, negative afterimages will appear as soon as the inducing lines are occluded by target elements. The “negative lens” targets used in previous studies (e.g., Anstis, 2012) are essentially optimal stimuli that mimic some aspects of naturally occurring negative afterimages. In Movie 2, when the target is absent, the afterimages give the impression that the physically empty rectangle contains some form of dynamic texture, although the structure of that texture is somewhat hard to describe. Further exploration of these dynamically released afterimages—and the spatial gradients they generate—may well yield interesting insights into furrow-type illusions and beyond. Here, however, we focus specifically on their role in creating partial object doubling.

**Movie 2.**
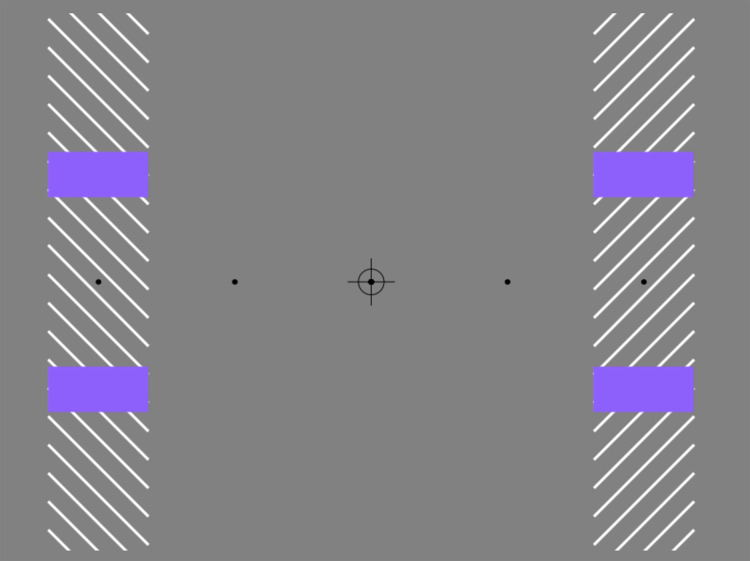
Negative afterimages and partial object doubling. https://maltacogsci.org/POD/POD_Movie2.html

In Movie 3, this role is more directly illustrated. Here, we have used “blocking” rectangles to “absorb” the released afterimages when they are on the leading edge of the outward-moving targets. During this phase of motion, no doubling occurs. However, when moving inwards, the afterimages are released as usual around the central shapes and doubling is experienced. Note that doubling occurs here in the absence of an apparent shift in the global trajectory of target objects.

**Movie 3.**
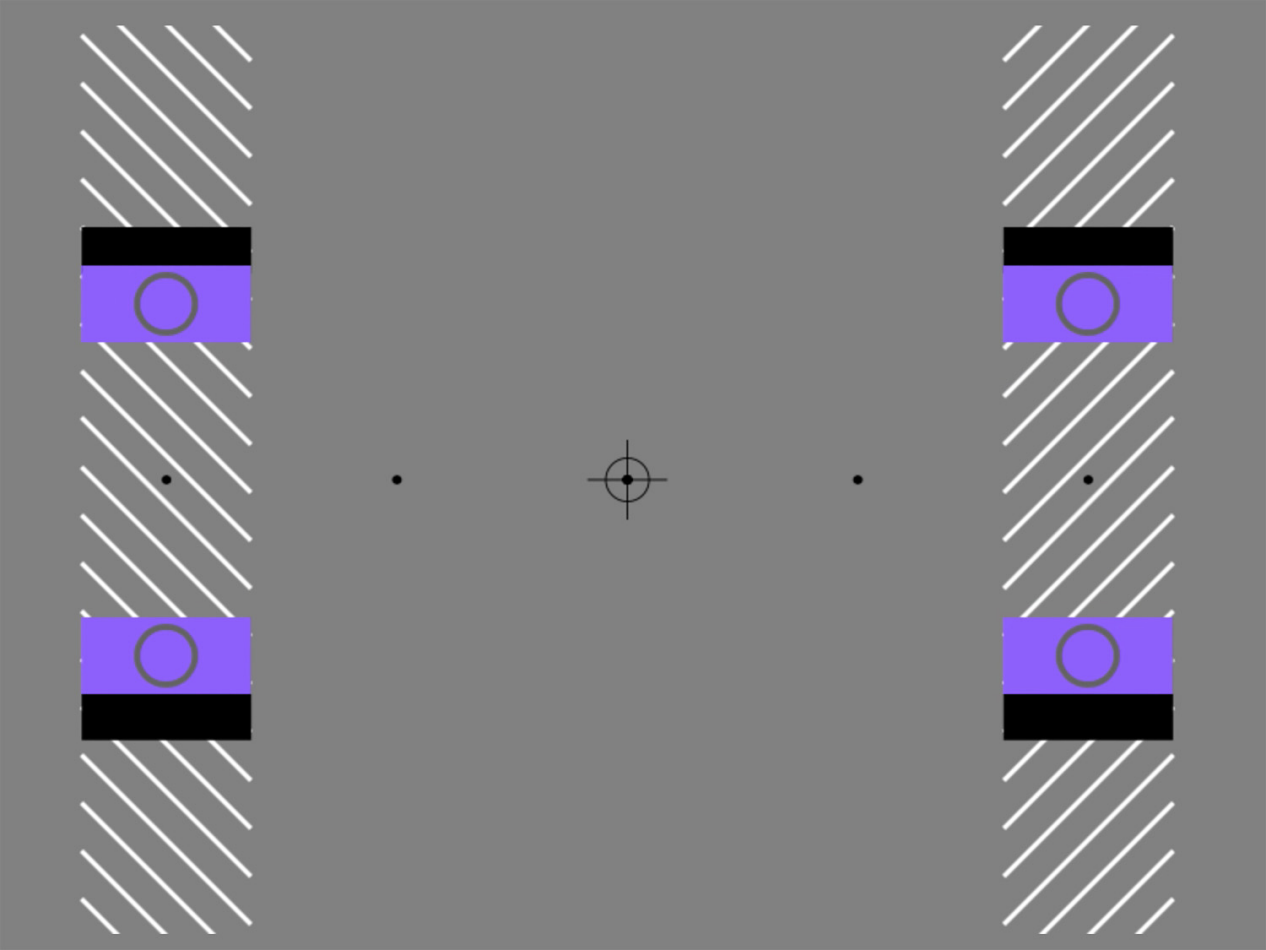
Blocking of negative afterimages disrupts partial object doubling. https://maltacogsci.org/POD/POD_Movie3.html

Movie 4 shows another display variant where doubling can be experienced when motion is perceived veridically. In this display, the white surround to the aperture provides such a strong global cue to motion direction that the illusory deviation is overridden. Nevertheless, the borders seem to amplify the release of the negative afterimages, and when the target shapes appear, there is still a clear, if perhaps slightly reduced, sense of doubling.

**Movie 4.**
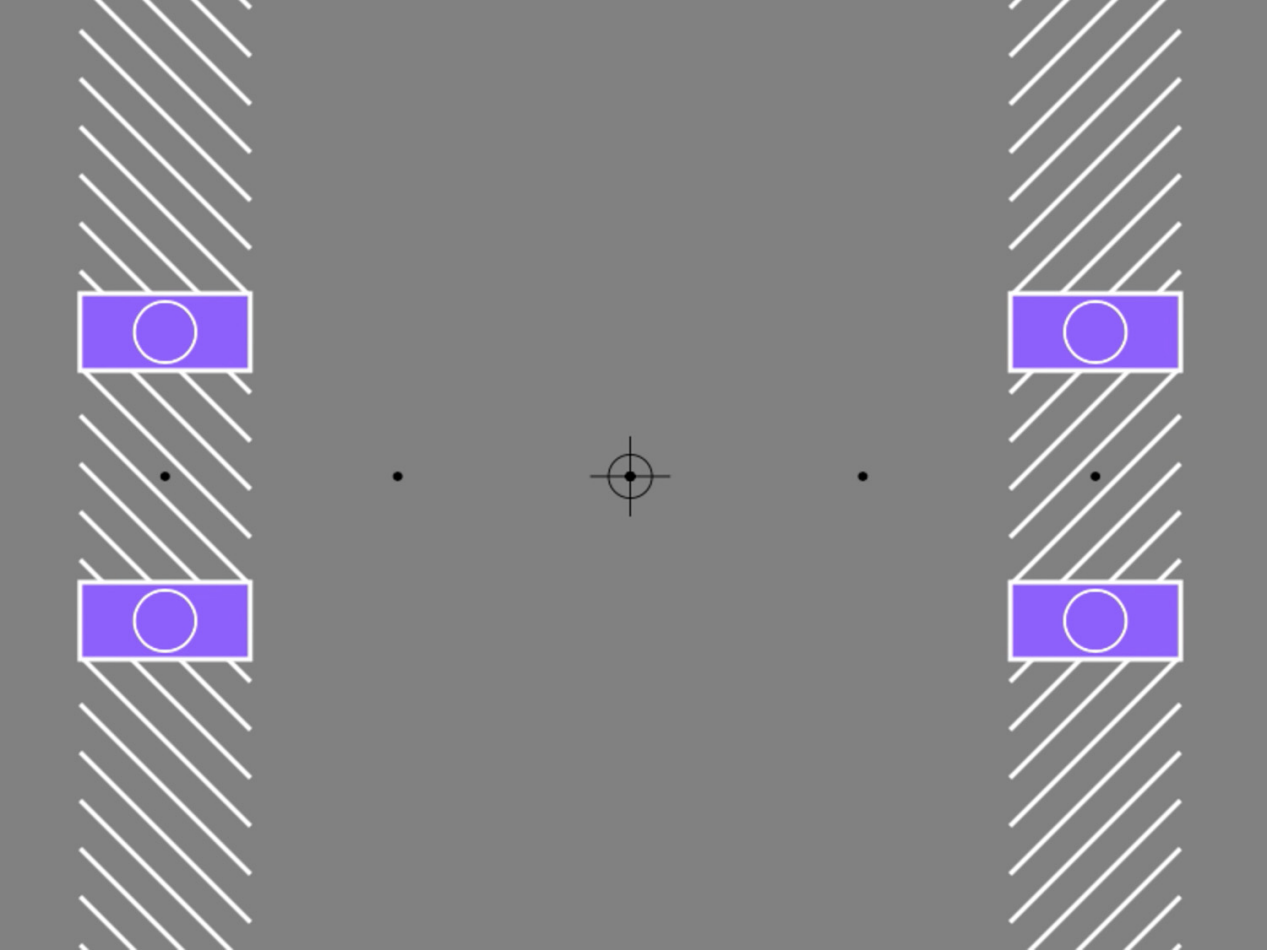
Partial object doubling without illusory motion. https://maltacogsci.org/POD/POD_Movie4.html

How do afterimages give rise to partial object doubling? One possible mechanism might be illusory conjunctions between the drawn shape elements and the afterimages. For example, if the weaker afterimage signals are hard to categorize in terms of their structure, they might “borrow” shape information from the more visible target elements leading to a form of confabulation that “smears” the overall object percept horizontally.

Another factor is almost certainly some form of motion-induced position shift (Whitney, 2002). The dynamically released afterimages—along with the line terminators at the edges of the rectangle—have a strong horizontal motion component, thanks to the aperture effect (Lorenceau & Shiffrar, 1992). Anstis (2012) had already noted the relationship between the furrow illusion and the barber pole effect. This within-aperture motion could cause a local, horizontal shift in the perceived position of the shape, either in the same (e.g., Whitney & Cavanagh, 2000) or the opposite (Duncker, 1929) direction to the afterimage motion.

As the vertical motion within the columns provides a strong global cue to position, the visual system has to resolve a conflict. As noted earlier, when computing perceived heading, it is thought that such conflicts are solved by taking a weighted vector average of local and global signals, giving rise to a single, often illusory percept (Anstis, 2012; Lisi & Cavanagh, 2015; Tse & Hsieh, 2006). Here, however, it seems that the visual system is not able to arrive at a single solution that merges local and global position information about the shape within the aperture, and partial object doubling occurs as the target is effectively pulled in two directions at once.

## Supplemental Material

**Figure:** Video 1.

**Figure:** Video 2.

**Figure:** Video 3.

**Figure:** Video 4.
